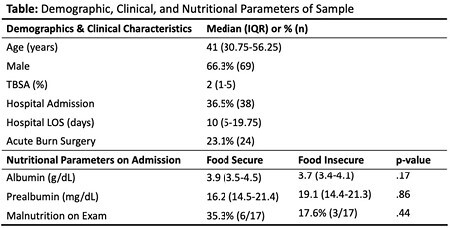# 59 Prevalence, Risk Factors, and Outcomes Associated with Food Insecurity in a Burn Clinic

**DOI:** 10.1093/jbcr/irae036.051

**Published:** 2024-04-17

**Authors:** Erin E Ross, Megan C Fobar, Laura Herrera Gomez, Justin Gillenwater, Haig A Yenikomshian

**Affiliations:** Keck School of Medicine of USC, Los Angeles, CA; Los Angeles General Medical Center, Los Angeles, California; University of Southern California, Los Angeles, California; Keck Medicine of USC, Los Angeles, CA; Keck School of Medicine of USC, Los Angeles, CA; Los Angeles General Medical Center, Los Angeles, California; University of Southern California, Los Angeles, California; Keck Medicine of USC, Los Angeles, CA; Keck School of Medicine of USC, Los Angeles, CA; Los Angeles General Medical Center, Los Angeles, California; University of Southern California, Los Angeles, California; Keck Medicine of USC, Los Angeles, CA; Keck School of Medicine of USC, Los Angeles, CA; Los Angeles General Medical Center, Los Angeles, California; University of Southern California, Los Angeles, California; Keck Medicine of USC, Los Angeles, CA; Keck School of Medicine of USC, Los Angeles, CA; Los Angeles General Medical Center, Los Angeles, California; University of Southern California, Los Angeles, California; Keck Medicine of USC, Los Angeles, CA

## Abstract

**Introduction:**

Adequate nutrition in burn care is paramount to wound healing. As a result, food insecurity may pose a significant risk to burn patients. Due to increased caloric need, burn patients may be at increased risk for food insecurity. Here, we assess food insecurity prevalence, risk factors, and possible associated complications of wound healing.

**Methods:**

Adult patients at a outpatient burn clinic answered the US Department of Agriculture Household Food Security Survey Module and questions about employment status and supplemental food resource use. Clinical data was abstracted from the medical record. Wound complication was defined as graft loss requiring regrafting or wound infection. Bivariate analysis by food security level was conducted by Chi-square, Fisher’s Exact test, or Mann-Whitney test. Association of age, race/ethnicity, total body surface area burned (TBSA), surgery count, hand or face burn, and unemployment to food insecurity were assessed by logistic regression, with significant predictors included in a multiple logistic regression model.

**Results:**

104 patients completed surveys, with 44.3% of patients experiencing food insecurity in the past 12 months. Only 48.9% of food insecure patients were using any supplemental resources for food access. Among 88 patients with complete healing data, wound complications in food secure and insecure patients were 21.3% and 38.7% respectively, (p= .57, power = 43%). Of the 22 patients with wound complications, 45% had diabetes (p=.022, Chi-square). In our final regression model, food insecurity was associated with TBSA (p=.045) and unemployment (p =.0075).

**Conclusions:**

Many patients are experiencing food insecurity during a time of increased metabolic need, and resources for food access are underutilized. Further research is needed to determine if food insecurity is associated with increased wound healing complications.

**Applicability of Research to Practice:**

Screening for food insecurity should be a routine component of outpatient burn care, and processes to connect patients to benefits for which they may be eligible are also needed.